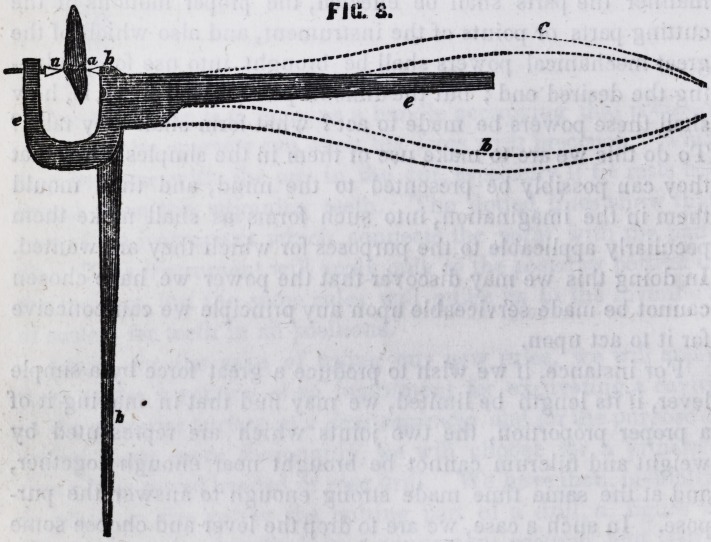# Contributions to Operative and Mechanical Dentistry

**Published:** 1845-12

**Authors:** W. H. Elliot


### ARTICLE VII.

### *Contributions to Operative and Mechanical Dentistry.*

By W. H. Elliot, D. D. S.

No. 7.

### HINTS TO THE PROFESSION ON THE INVENTION AND CONSTRUCTION OF DENTAL INSTRUMENTS.

Although much may be found in the practice of every enlightened member of the profession, that is universally received as correct and useful, there may also be something discovered which would be considered by many practiciens as unscientific and injurious; this fact alone would be sufficient evidence, if we had no other proof, that our noble science is far from enjoying that state of perfection that its importance seems to demand. But while we witness in our daily practice the uncertain manner in which the dentist proceeds to arrive at some of the most simple ends—the necessity for scores of instruments that have never yet received form, even in the imagination, the conviction comes upon us with still greater force.

There are yet too many operations that may justly be found fault with, either in the method in which the operation is performed, or the principle upon which the instrument acts. It is true, that there are a few instruments that may not appear susceptible of any improvement to the most prolific inventive genius, while there are others that seem as well adapted to one operation as another, being totally unsuited to any. In the midst of the most trivial operation, we are often at a loss for an instrument with which to proceed, and at such times, we are forced to feel that there is some principle entirely wanting, some link in the chain of instruments that has never yet been forged. While so much remains to be done, no apology can be offered for a moment's idleness, but we should rather consider it a privilege to assist in stamping the present age as one of improvement in our own profession, to add a single spark to "the blaze of the nineteenth century." But the responsibility rests not entirely with inventive minds, they are generally ready to exercise their talents when an opportunity offers, and they may receive much valuable assistance from writers if they will take the trouble to suggest the necessity for any instrument they may think required, and, if while they are praising the virtues of some instruments, they will mention the faults of others, so that they may be corrected, they will confer a greater benefit. If any one doubts the propriety of this, let him make the experiment by calling for an instrument for any operation, mentioning all the advantages he wishes it to possess, and we doubt not if it be not a solecism in itself it will be immediately produced.

We do not expect in the present instance to lay down rules by which every man may become an inventor; that would indeed be a field too ample for our limited capacities; such a work, if the proposition were possible, would fill volumes like the one in which this article is published. Our highest aim is to render some slight assistance to those who already possess the power of conceiving new causes, but are entirely without any fixed principles upón which to act. Believing, as our friend Bishop Hopkins once said to us, that "the gift of invention is the gift of heaven, he who possesses it cannot live without exercising it, if he would; but he who does not possess that faculty, *may* be capable of accomplishing almost any object; he may fly, but he cannot invent." The inventive mind may be systematized, but it cannot be cultivated, it may be taught to act upon specified principles which will enable it to overcome, to a certain degree, the opposing difficulties, to make use of what is already known in the discovery of things unknown; but it must ever labor with the same embarrassment in the search of new principles.

Without the ability of fixing the attention upon one subject for a considerable length of time, little advancement can be made in mechanical discoveries; for in the very commencement we must find out and retain the principle upon which the machine is to act without the use of drawings, or even the assistance of fancied forms; these would only encumber the mind until the peculiar motions required and the means of producing those motions has been decided upon.

Principles and not things must first engage the attention; the recollection of forms, which must be constantly changing, can be of no service. For instance, if we wish to invent an instrument for extracting the teeth, we are first to consider its point of contact upon the tooth, the principle which is to retain it there, the position of the fulcrum, if one be used, the motion which is to disengage the tooth, the means of producing that motion, the effect of the necessary force upon the jaw, &c.; and if these all be satisfactory, we are then prepared to give form and proportion, first to the moving parts, and lastly to the basis, or foundation of the instrument, which is to hold the moving parts in their relative positions. As well might the poet express a sentiment before he has conceived it, or the painter draw the outlines before he has decided what he will portray, as the inventor, to give so much as an imaginary form to the parts of a machine before he has found a suitable principle for it to act upon.

We have observed, in beginners particularly, that the mind constantly reverts to forms, a total inability to consider the motions of a machine without giving to the parts that make those motions a fancied shape, and thus by materializing every thought, the whole power of the mind is concentrated in mere remembrance of figures, with which at first it has nothing to do.

Unencumbered, the mind passes rapidly from one principle to another, until it hits upon one suited to its present necessities. Motions and not matter is what we have to deal with first, and when we have learned to think of a lever, a crank, or a wheel, and follow their several motions perfectly without connecting them with any thing material, we shall have taken the first indispensable and most important step in training the mind to mechanical discoveries. Then, and not till then, can we proceed independently of what is already known in mechanics.

Many inventions have been made by comparison, or in other words, by applying old principles to new purposes, as in case of the reacting steam engine, which was taken up and applied to the propelling of saw-mills, more than two thousand years after it was invented and used by the ancients in performing their religious rites, and is said to have been made in imitation of the reacting water-wheel; but such improvements are seldom the product of an inventive mind, nor are they often of much utility, since a slight change in the nature of the material wrought, or in the element in which they act, (as was the case with the machine mentioned above,) is liable to defeat the experiment; therefore, time spent in search of a simile by which to fashion another machine for a new purpose is lost; for although a design may operate to perfection when applied to the purposes for which it was invented, it will generally be found full of faults, both in principle and construction, when applied under different circumstances to new ends.

The pile, which is easily driven into the earth by the falling weight, would cripple under the application of a lever power of equal or of a much smaller force, and yet the power of a lever is found indispensably necessary in some instances where the principle of the pile-driver would be entirely out of place. In the invention and construction of dental instruments and other apparatus, there is the same liability to misapplication of principle, an example of which may be found in the hammer and punch for *pressing* fillings, or in the use of the smith's vice for *striking* up plates.

Nothing short of a thorough knowledge of mechanical causes and effects, can enable the inventor to choose correctly when he is searching for new principles.

In the invention of instruments of simple construction, that is, such as need not necessarily consist of more than one piece, after we have decided upon its peculiar motions, and the manner in which it is to effect the parts operated upon, we may then give form to the cutting part or point, mentally, and fix it in the position it will occupy when in use. We may then imagine a handle of suitable size and length, placed in such a position that if it were connected with the point, this might receive from it the necessary force and direction without inconvenience to the operator or risk of accident. We have then only to join the point with the handle in such a way that the connecting part will not interfere with the motions of the instrument.

For instance, if we wish to produce an instrument for removing tartar from the posterior surfaces of the lower *incisors*, our first step is to look about for a principle, which may readily be found in the instruments in use at the present day, viz. an upward motion of a thick, firm edge, used as a scraper. Our second step is to imagine a cutting edge of proper shape and dimensions, placed against that part of the tooth from which the tartar is to be removed, *a*, figure 1, represents the tooth, and *b*, the point in its proper position.

FIG. 1.

FIG. 2.

Our third step is to imagine a handle occupying that position in which the operator can exert his force with precision. This may be done with the one in the cut, especially if he rests his thumb upon the adjoining teeth. The dotted lines show that part of the instrument which connects the point with the handle. This instrument will apply only to the posterior portion of the incisors, but the same rules will guide us in the invention of scalers for teeth in all positions.

Again, for the sake of trying our new rules, we will apply them to the invention of an instrument for excavating a cavity in the posterior surface of a *dens sapientia*, and for the purpose of trying them more thoroughly, we will choose for a principle that of the round headed or rose drill. We have then, mentally, to place in the cavity the cutting part of a drill, *a*, figure 2. Then place the handle *b* in a convenient position, and lastly, the connecting part, *c*. The only objection that can be offered to this instrument is, that it will not perform an entire revolution, which if it be a fault, cannot be remedied in an instrument of a simple construction, but may be readily overcome by giving to the instrument the complex form.

Complex instruments are those in which it becomes necessary to obtain, by means of some of the mechanical powers, a greater amount of force or variety of motion than can conveniently be given it by the hand ; such are the excising instrument, bow, drill, &c. Instruments of this character generally consist of several moving parts, and a basis whose office it is to hold the moving parts in their relative positions.

The invention of complex instruments is an undertaking of a more difficult nature, and yet all the instructions that can be given on that subject may be communicated in a very few words. In the first step we have not only to decide upon the manner in which the instruments shall affect the parts, the necessary motions, but also the means of producing those motions.

The second step exhibits the inventive genius in all its force and beauty; it is comparatively an easy matter to decide what manner the parts shall be effected, the proper motions of the cutting parts or points of the instrument, and also which of the great mechanical powers shall be brought into use for producing the desired end ; but the difficult point to be solved, is, how shall these powers be made to act? what form shall they take? To do this we are to make use of them in the simplest form that they can possibly be presented to the mind, and then mould them in the imagination, into such forms as shall make them peculiarly applicable to the purposes for which they are wanted. In doing this we may discover that the power we have chosen cannot be made serviceable upon any principle we can conceive for it to act upon.

For instance, if we wish to produce a great force by a simple lever, if its length be limited, we may find that in making it of a proper proportion, the two joints which are represented by weight and fulcrum cannot be brought near enough together, and at the same time made strong enough to answer the purpose. In such a case, we are to drop the lever and choose some other power. A difficulty of this description is presented in the common excising forceps. If the centre of the joint of this instrument could be brought within two lines of the cutting edges, the operator could use it with much greater precision, and less risk of injury to the parts by the violence of the effort ; this difficulty, may, however, be overcome by changing the principle upon which the lever acts; and for example, we will take the excising instrument. In this instance, our only object is to so alter the principle and proportion of the lever, as to produce an instrument of much greater power than the one in general use.

In the first place then, the tooth must be cut off by the approximation of two firm edges, moved by the simplest of all means, a lever. The next step is to fix, in the mind's eye, the cutting points of the instrument in their place against the tooth, and also to place a lever so as to bear against one of the points, *aa*, figure 3, the cutting points, *bb*, the lever in its simplest form. Thirdly, we must add a basis or foundation that will hold one point and the fulcrum of the lever at a fixed distance from each other, and also serve as a handle for the instrument, *cc*, represents the basis.

FIG. 3.

We now have an instrument which is mechanically correct, though not by any means a convenient one. As it is, it requires both hands of the operator to use it, but by bringing the lever near the handle, it may be used with one hand. The dotted lines show the alteration made for the sake of convenience.

In the whole arrangement of the instrument, regard should be had to the nature of the force required. If it be necessary to make a powerful effort, and at the same time to have the extent of the motion perfectly at the control of the operator, though the opposing force might by accident be removed suddenly, it is necessary to have the instrument so arranged as to bring into action the *flexor et extensor carpi ulnaris*, this is only necessary in extracting teeth with the key. When teeth are extracted in this manner, the shaft of the instrument forms a right angle with the forearm. If the forearm be brought into a line with the shaft of the instrument so as to call into action the *pronator* or *supinator* muscles, the operator has not sufficient control over their motion, so that if the tooth lose its attachment to the jaw suddenly, the force being still continued, it passes out laterally, carrying with it a portion of the alveolus, or leaving one of its fangs behind, where, if the force of the instrument had been discontinued at the proper time, the operation might have been safely completed with the forceps.

In case a rapid succession of motions be required, the flexors and extensors of the wrist may properly be used, since their action may be continued much longer without fatigue than any other muscle about the hand or forearm, in proof of which the bow-drill and file furnish examples.

If a sudden effort be required like that necessary to excise a large tooth, the *pronator* muscle is best suited to the purpose, for in that case no injury can come from continuing the motion too far.

---

## Figures and Tables

**FIG. 1. f1:**
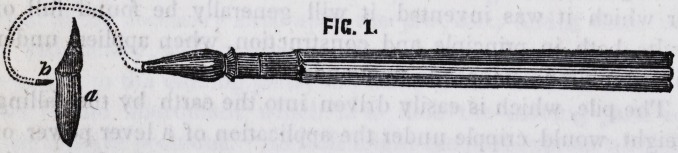


**FIG. 2. f2:**
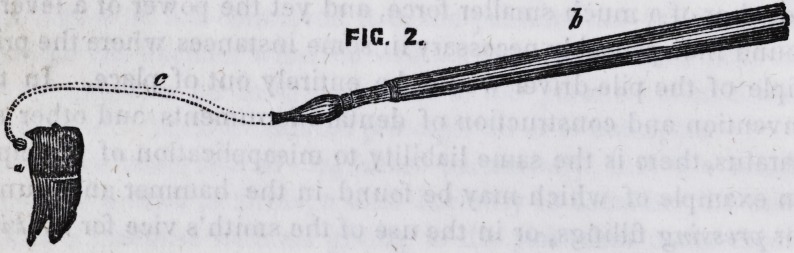


**FIG. 3. f3:**